# Dietary zinc supplementation rescues fear-based learning and synaptic function in the *Tbr1*^+/−^ mouse model of autism spectrum disorders

**DOI:** 10.1186/s13229-022-00494-6

**Published:** 2022-03-18

**Authors:** Kevin Lee, Yewon Jung, Yukti Vyas, Imogen Skelton, Wickliffe C. Abraham, Yi-Ping Hsueh, Johanna M. Montgomery

**Affiliations:** 1 Department of Physiology and Centre for Brain Research, Faculty of Medical and Health Sciences, University of Auckland, 85 Park Road, Grafton, Auckland, 1023 New Zealand; 2 Present Address: INSERM, Neurocentre Magendie, U1215 Bordeaux, France; 3 Department of Psychology and Brain Health Research Centre, University of Otago, Dunedin, New Zealand; 4 Institute of Molecular Biology, Academia Sinica, 128, Section 2, Academia Rd., Taipei, 11529 Taiwan

**Keywords:** Autism spectrum disorder, T-brain-1, Dietary zinc supplementation, Amygdala, Glutamatergic synapses, *N*-methyl-d-aspartate receptors

## Abstract

**Background:**

Autism spectrum disorder (ASD) is a neurodevelopmental disorder characterised by a dyad of behavioural symptoms—social and communication deficits and repetitive behaviours. Multiple aetiological genetic and environmental factors have been identified as causing or increasing the likelihood of ASD, including serum zinc deficiency. Our previous studies revealed that dietary zinc supplementation can normalise impaired social behaviours, excessive grooming, and heightened anxiety in a *Shank3* mouse model of ASD, as well as the amelioration of synapse dysfunction. Here, we have examined the efficacy and breadth of dietary zinc supplementation as an effective therapeutic strategy utilising a non-*Shank*-related mouse model of ASD—mice with *Tbr1* haploinsufficiency.

**Methods:**

We performed behavioural assays, amygdalar slice whole-cell patch-clamp electrophysiology, and immunohistochemistry to characterise the synaptic mechanisms underlying the ASD-associated behavioural deficits observed in *Tbr1*^+/−^ mice and the therapeutic potential of dietary zinc supplementation. Two-way analysis of variance (ANOVA) with Šídák's post hoc test and one-way ANOVA with Tukey’s post hoc multiple comparisons were performed for statistical analysis.

**Results:**

Our data show that dietary zinc supplementation prevents impairments in auditory fear memory and social interaction, but not social novelty, in the *Tbr1*^+/−^ mice. *Tbr1* haploinsufficiency did not induce excessive grooming nor elevate anxiety in mice. At the synaptic level, dietary zinc supplementation reversed α-amino-3-hydroxy-5-methyl-4-isoxazolepropionic acid receptor (AMPAR) and *N*-methyl-d-aspartate receptor (NMDAR) hypofunction and normalised presynaptic function at thalamic-lateral amygdala (LA) synapses that are crucial for auditory fear memory. In addition, the zinc supplemented diet significantly restored the synaptic puncta density of the GluN1 subunit essential for functional NMDARs as well as SHANK3 expression in both the basal and lateral amygdala (BLA) of *Tbr1*^+/−^ mice.

**Limitations:**

The therapeutic effect of dietary zinc supplementation observed in rodent models may not reproduce the same effects in human patients. The effect of dietary zinc supplementation on synaptic function in other brain structures affected by *Tbr1* haploinsufficiency including olfactory bulb and anterior commissure will also need to be examined.

**Conclusions:**

Our data further the understanding of the molecular mechanisms underlying the effect of dietary zinc supplementation and verify the efficacy and breadth of its application as a potential treatment strategy for ASD.

## Background

Autism spectrum disorder (ASD) is a prevalent neurodevelopmental disorder characterised by social and communication deficits, excessive repetitive behaviours, sensory abnormalities, and cognitive dysfunction [1, 2]. Although the full understanding of the aetiology of ASD has not yet been established, a diverse range of genetic aberrations and environmental factors are thought to contribute to the heterogeneity of behavioural traits observed in children with ASD [3]. In recent years, next-generation whole-genome sequencing has identified hundreds of deleterious variants in 10–30% of ASD patients [4, 5]. Among the recurrent ASD-associated high-confidence risk genes, *T-brain-1* (*TBR1*) has been of particular interest because it encodes a neuron-specific transcription regulator of the T-box family that modulates cortical development and brain wiring [6]. To date, genetic aberrations in one allele of *TBR1* have been discovered in ASD patients who commonly display socialisation impairments, defective language skills, and intellectual disability [7–13]. Consistently, mouse models of *Tbr1* haploinsufficiency (*Tbr1*^+/−^) or human *TBR1* mutation knock-in (*Tbr1*^+*/K228E*^) also display ASD-like behavioural deficits [14–16], and thus, serve as good models to further our understanding of the pathogenic mechanisms of ASD induced via de novo* TBR1* mutations.

*Tbr1* is highly expressed in early-born excitatory glutamatergic neurons [17–19], and plays a critical role in neuronal differentiation, migration, laminar fate, and regional identity during brain development [20–27]. Mice carrying a homozygous mutation of the *Tbr1* gene (i.e. deletion of both alleles of *Tbr1*, *Tbr1*^*−/−*^) demonstrate impaired differentiation and migration of embryonic cortical neurons as well as abnormal axonal projections in the cerebral cortex, amygdalae, and olfactory bulb [14, 18, 20, 23], resulting in early postnatal death [18]. *Tbr1*^+/−^ mice display significant diminishment of the anterior commissure (a bundle of white matter tract fibres connecting two cerebral hemispheres, including the two amygdalae) [14], which is also observed in individuals with de novo* TBR1* variants [28].

In addition to its role in brain development, *Tbr1* also responds to changes in neuronal activity. Enhanced neuronal excitation upregulates *Tbr1* expression in cultured mature neurons [29], and its direct interaction with calcium/calmodulin-dependent serine protein kinase (CASK), a multidomain scaffolding protein associated with neural development and synaptic function [30–33]. Together with the CASK interacting nucleosome assembly protein (CINAP) [6], TBR1, CASK, and CINAP form a tripartite complex that regulates *Grin2b* expression [29, 34, 35]. As the upregulation of *Tbr1* expression requires activation of calcium/calmodulin-dependent protein kinase II (CaMKII) via calcium influx through NMDARs [29], *Tbr1* and *Grin2b* form a modulatory feedback loop that links neuronal activation and synaptic function. This is evident in *Tbr1* deficient mice where *Grin2b* expression is decreased [36], and amygdalar neurons of *Tbr1*^+/−^ mice fail to show neuronal activation-induced upregulation of *Grin2b* expression and impaired NMDAR function at thalamic-amygdalar synapses [14, 37–39].

d-cycloserine, a partial agonist of NMDARs, has been examined as a therapeutic agent in *Tbr1*^+/−^ mice to correct the reduced neuronal activation and NMDAR hypofunction that are proposed to be the pathogenic cellular mechanisms underpinning ASD-like behaviours [14, 15]. Systemic application or local infusion of d-cycloserine effectively restored neuronal activation in both amygdalae and olfactory bulb and reversed ASD-like behaviours induced by *Tbr1* haploinsufficiency [14, 15]. Interestingly, trans-synaptic zinc mobilisation via clioquinol, a zinc chelator and ionophore, has also been shown to successfully rescue social interaction deficits and NMDAR hypofunction in *Tbr1*^+/−^ mice [37]. Zinc is a prevalent metal in the brain that modulates synaptic function and plasticity via interaction with neuronal ion channels, transporters, receptors, and synaptic structural proteins [40]. Increased zinc levels have been shown to promote synaptic protein scaffold formation, facilitating synapse development and maturation [41–44]. We have previously shown that dietary zinc supplementation for 6 weeks post-weaning can prevent ASD-associated behavioural deficits and adjust the structure and function of glutamatergic cortico-striatal synapses in *Shank3* deficient mice [45]. Here, we have evaluated the therapeutic breadth of dietary zinc supplementation beyond ASD-linked *Shank3* mutations by examining its effectiveness in *Tbr1*^+/−^ mice. Specifically, we show that dietary zinc elevation can restore social interaction, fear memory, and functional thalamic-amygdalar synapses in the *Tbr1*^+/−^ mice, thereby broadening the applicability of dietary zinc supplementation for ASD treatment.

## Methods

### Animals

All animal manipulations and experiments were performed under regulations approved by the University of Auckland Animal Ethics Committee and in adherence to the ARRIVE guidelines. The *Tbr*^+/−^ mice were provided by Y.-P. Hsueh (Institute of Molecular Biology, Academia Sinica, Taipei, Taiwan) [14, 15] with permission of Dr. John Rubenstein (University of California, San Francisco) and maintained at the Vernon Jansen Unit animal facility at the University of Auckland, Auckland, New Zealand. Wildtype *Tbr*^+*/*+^ (WT) and heterozygous *Tbr*^+/−^ mice were generated from *Tbr*^+/−^male x WT female breeding pairs. All experimental animals were housed under a standard 12/12-h light–dark cycle. Offspring were ear-punched for genotyping and identification at postnatal day 10, and were kept with the dam until weaning at postnatal day 21. After weaning, animals were housed in groups of 2–4 per individually ventilated cage with mixed genotypes. Food and water were available ad libitum.

### Experimental animals

At weaning, animals were randomly assigned to a control zinc diet (30 ppm [parts per million] zinc; D19410B; Research Diets, Brunswick, NJ, USA) or a zinc supplemented diet (150 ppm zinc; D06041101; Research Diets, Brunswick, NJ, USA), for 6–8 weeks. These egg white-based diets were identical in composition except for their zinc levels. As we have previously shown in *Shank3*^*ex13–16−/−*^ mice [45, 46], which have the same C57BL/6/J background, no adverse effects on animal health, weight, or development were evident on either diet. No significant differences were observed in body weight for all *Tbr1* genotypes: Male mice at 9 weeks: WT_30ppm_, 22.00 ± 0.53 g; *Tbr*^+/−^_30 ppm_, 21.00 ± 0.65 g; WT_150ppm_, 22.00 ± 0.66 g; *Tbr*^+/−^_150 ppm_, 21.82 ± 0.99 g. Female mice at 9 weeks: WT_30ppm_, 20.00 ± 0.51 g; *Tbr*^+/−^_30 ppm_, 19.75 ± 0.41 g; WT_150ppm_, 19.70 ± 0.40 g; *Tbr*^+/−^_150 ppm_, 20.09 ± 0.46 g. Behavioural, electrophysiological, and imaging experiments were performed at 9–11 weeks of age on male and female mice on either diet. In total 82 WT mice and 88 Heterozygous mice were used in these experiments. Both genders were included in all experimental data sets, and WT mice were used as controls (WT: immunocytochemistry—5 males and 5 females; electrophysiology—6 males and 7 females; behavioural experiments—27 males and 22 females; *Tbr*^+/−^: immunohistochemistry—5 males and 5 females; electrophysiology—5 males and 9 females; behavioural experiments—25 males and 23 females). The animals were randomly grouped and were used independently for behavioural, electrophysiological, or imaging experiments. All experiments and analyses were conducted with the experimenter blinded to genotype and zinc diet by independent animal coding with a unique identification number at weaning.

### Behavioural tests and analysis

All behavioural tests were performed in an isolated room under the light cycle, and the animals were habituated in the room for at least one hour before testing. Except for the auditory fear conditioning test, a ceiling camera DFK21AF04 (SDR Scientific) was used for recording and monitoring animal behaviour. EzTrack (Open source) [47] was used for generating heat maps and tracking animal movement. The grooming test arena, dark–light chamber, and three chamber apparatus were washed with 70% ethanol and then 3% acetic acid between each behavioural trial. The same cohort of animals underwent the grooming, light and dark, social interaction, and social novelty tests, respectively, over a period of 1–2 weeks. No two behavioural tests were performed on the same day. A separate cohort was used for the auditory fear conditioning test so that social behaviour was not influenced by fear conditioning.

#### Auditory fear conditioning test

Auditory fear conditioning was performed in operant chambers (17 × 17 × 25 cm^3^, Ugo Basile) within sound-attenuating boxes (Ugo Basile) illuminated by a 7.5 W white light and constant white noise of 60 dB playing in the background through built-in speakers. Animal motion was recorded with the built-in camera, and the freezing behaviour was analysed by EthoVision XT Version 12.0 (Noldus). The chambers were cleaned with 3% acetic acid (training days and day 0) or 70% ethanol (day 1). Each mouse was placed in the operant chamber and habituated for 8 min on training day. On day 0, each mouse was placed into the operant chamber for 4 min, and then 3 auditory conditioned trials were performed, comprising of a tone (2 kHz; 80 dB; 18 s) followed by an electric foot shock (0.6 mA; 2 s) in 1 min intervals. Freezing responses of the mice immediately after the third electric foot shock over 60 s were measured as “after shock”. Mice were returned to their home cages after the training. On day 1, mice were placed in a novel operant chamber, and forty tones (80 dB; 2 kHz; 20 s) were applied at 5 s intervals. Time spent freezing was averaged over the first ten auditory stimulations [14].

#### Grooming behaviour

Under red light conditions [15 lx], each mouse was placed in a cylindrical arena [17 cm radius] and habituated for 10 min. The behaviour of the mouse was then recorded, and the grooming behaviour was analysed over 30 min. Grooming behaviour included licking, wiping, and rubbing any body parts [45, 46].

#### Dark–light emergence test

Each mouse was placed in a dark chamber (001 lx) for 5 min. The door between the dark and light chambers was opened enabling the mouse to freely explore the chambers for 10 min. The latency to enter the light chamber (275 lx), transition number to enter the light chamber, and the time spent in the light chamber were measured.

#### Three-chamber social interaction test

The three chamber apparatus was used under low light conditions (15 lx) and conducted as previously described [14, 37, 45, 46]. Each mouse was placed in the middle chamber, and a mesh container was placed in both the left and right chambers. The doors between the chambers were then opened to enable the mouse to explore all three chambers. After 10 min, the mouse was gently guided to the centre chamber, and the doors were closed. In phase 2, a sex and age-matched stranger mouse (Stranger 1: S1) was placed in one of the containers, while the other chamber’s container remained empty (Empty cup: E). The test mouse was then allowed to explore freely for 10 min. The mouse was again gently guided to the centre chamber, and the doors were closed. In the last phase of the test, another sex and age-matched stranger mouse (Stranger 2: S2) was introduced in the previously empty container. The mouse was then allowed to explore for 10 min in all three chambers. The social interaction reference index was calculated by the time spent in close interaction with S1 subtracted by time spent in close interaction with the empty cup in the chamber. The social novelty reference index was calculated by the time spent in close interaction with S2 subtracted by time spent in close interaction with S1.

### Electrophysiology

#### Acute brain slice preparation

Acute coronal brain slices were prepared from 9- to 11-week-old mice, as described previously [37]. Mice were euthanised with CO_2_ and decapitated, and the brain was removed and transferred to carbogenated (95% O_2_, 5% CO_2_) ice-cold (6–8 °C) protective cutting artificial cerebrospinal fluid (aCSF): (in mM), 93 N-methyl-d-glucamine (NMDG), 2.5 KCl, 1.25 NaH_2_PO_4_, 30 NaHCO_3_, 20 HEPES, 25 glucose, 2 thiourea, 5 L-ascorbic acid, 3 Napyruvate, 0.5 CaCl_2_, 10 MgSO_4_.7H_2_O, pH 7.4, 295–305 mOsm. The brain was rapidly sectioned at 300 µm thickness using a vibratome (VT1200S, Leica Biosystems; 1 mm x-plane blade vibration, 0.05 mm/min y-plane advancement speed, 0 mm z-axis vibration calibration), obtaining on average 3–4 coronal slices including the lateral amygdala (LA). Slices were transferred to protective cutting aCSF at 34 °C for 12 min. Before recording, slices were maintained at room temperature (RT, 24 °C) for a minimum of 1 h, submerged in a holding chamber containing carbogenated recovery aCSF: (in mM) 97 NaCl, 2.5 KCl, 1.2 NaH_2_PO_4_, 30 NaHCO_3_, 25 glucose, 20 HEPES, 2 CaCl_2_, 2 MgSO_4_.7H_2_O, 2 thiourea, 5 L-ascorbic acid, 3 Napyruvate, pH 7.4, 295–305 mOsm. Slices were utilised for a maximum of 6 h from preparation, and any damaged, unhealthy slices were omitted from electrophysiological recordings.

#### Whole-cell patch-clamp recordings

Only brain slices with clearly defined thalamic afferents crossing the dorsolateral region of the LA, a converging location of auditory and somatosensory inputs, were utilised for whole-cell patch-clamp experiments. Slices were transferred to the recording chamber, superfused with carbogenated recording aCSF: (in mM) 119 NaCl, 2.5 KCl, 1 Na_2_HPO_4_, 1.3 MgSO_4_, 26.2 NaHCO_3_, 11 D- ( +)-glucose, 2.5 CaCl_2_, pH 7.4, 305–310 mOsm at RT at 2–3 mL/minute. The principal neurons of the LA were visualised using infrared differential interference contrast (IR-DIC) optics with a 40× water immersion objective lens mounted on an Olympus BX‐51 microscope (Olympus Corporation, Japan). A platinum-iridium concentric bipolar stimulating electrode was placed onto the thalamic afferent pathway to induce presynaptic stimulation of the principal neurons of the LA. Stimulation was performed with a constant isolated current stimulator (Model DS3; Digitimer, USA) with a duration of 500 μs at 0.05 Hz frequency. Whole-cell patch-clamp recordings of LA principal neurons were acquired with glass recording electrodes (borosilicate tubing filamented glass electrodes, BF150–86–7.5; Sutter Instrument Company, USA) of 5–7 MΩ resistance pulled by a vertical electrode puller (PC-10; Narishige, Japan), and filled with an internal solution: (in mM) 120 Cs gluconate, 40 HEPES, 5 MgCl_2_, 2 NaATP, 0.3 NaGTP and 5 QX314, pH 7.2, 298 mOsm. The distance between the stimulating electrode and recording electrode was approximately 200 μm. Electrophysiological signals were amplified (Multiclamp 700B; Axon Instruments, USA) and digitised (Digidata 1550B; Axon Instruments, USA). Events were sampled at 20 kHz and low-pass filtered at 2 kHz. All data were obtained and analysed using pClamp 10 acquisition software and Clampfit 10, respectively (Axon Instruments, USA). Series resistance (Rs) was < 25 MΩ. Rs was monitored before and after each recording paradigm, and any recordings with Rs variation greater than 20% were discarded from the analysis. Overall, we observed no differences across experimental groups in membrane capacitance (Cm: WT_30ppm_, 181.4 ± 7.607 pF; *Tbr*^+/−^_30 ppm_, 196.4 ± 15.03 pF; WT_150ppm_, 171.8 ± 8.396 pF; *Tbr*^+/−^_150 ppm_, 195.4 ± 8.585 pF; p > 0.05), membrane resistance (Rm: WT_30ppm_, 376.8 ± 33.38 MΩ; *Tbr*^+/−^_30 ppm_, 332.3 ± 52.92 MΩ; WT_150ppm_, 424.5 ± 53.06 MΩ; *Tbr*^+/−^_150 ppm_, 378.9 ± 26.02 MΩ; p > 0.05), or access resistance (Ra: WT_30ppm_, 20.85 ± 0.645 MΩ; *Tbr*^+/−^_30 ppm_, 22.62 ± 0.575 MΩ; WT_150ppm_, 20.88 ± 0.765 MΩ; *Tbr*^+/−^_150 ppm_, 21.33 ± 0.794 MΩ; p > 0.05).

To measure α-amino-3-hydroxy-5-methyl-4-isoxazolepropionic acid receptor (AMPAR)-mediated excitatory postsynaptic currents (EPSCs), LA principal neurons were voltage-clamped at – 70 mV and stimulated with an intensity that was adjusted to provide a half-maximal amplitude. AMPAR-mediated EPSCs were measured for 30 sweeps at 0.05 Hz. Only the peak amplitudes of monosynaptic EPSCs relative to the pre-stimulation baseline were used for data analysis. For paired-pulse ratio (PPR) and NMDAR/AMPAR ratio experiments, the stimulation intensity was modified to the level that stably evoked 200–350 pA AMPAR-mediated EPSCs. For paired-pulse stimulation, two consecutive stimuli separated by 50 ms were delivered to LA principal neurons (voltage-clamped at − 70 mV, 30 sweeps, 0.05 Hz). Only recordings that displayed stable evoked first EPSCs were included for data analysis. PPR was determined by dividing the peak amplitude of the second AMPAR-mediated EPSC response by that of the first peak current amplitude. For NMDAR/AMPAR ratio experiments, LA principal neurons were first voltage-clamped at – 70 mV, and 30 consecutive AMPAR-mediated EPSCs at a stimulus intensity that stably evoked 200–350 pA current responses were recorded (0.05 Hz) to ensure consistent presynaptic stimulation and AMPAR-mediated responses when measuring NMDAR EPSCs. Then, 10 μM CNQX was bath applied to block AMPAR-mediated current responses, and the holding potential was switched to + 40 mV to record NMDAR-mediated EPSCs (30 sweeps, 0.05 Hz). The NMDAR/AMPAR ratio was calculated as the average peak amplitude of NMDAR-mediated EPSCs divided by the average peak amplitude of AMPAR-mediated EPSCs. Picrotoxin (100 µM) was included in the recording aCSF to block GABA_A_ receptor-mediated inhibitory currents during stimulation. Decay kinetics of the NMDAR-mediated responses were measured by fitting a double exponential function to the decay phase of normalised NMDAR-mediated EPSCs (Clampfit 10) [46, 48]. To compare decay times between different experimental groups directly, we calculated a weighted mean decay time constant: *τ*_*w*_ = *τ*_*s*_[*I*_*s*_/(*I*_*f*_ + *I*_*s*_)] + *τ*_*f*_ [*I*_*f*_/(*I*_*f*_ + *I*_*s*_)], where *I*_*f*_ and *I*_*s*_ represent the amplitudes of the fast and slow decay components, respectively, and *τ*_*f*_ and *τ*_*s*_ represent fast and slow decay time constants, respectively.

### Luxol fast blue staining

After dietary supplementation with normal (30 ppm) and increased (150 ppm) zinc for 6–8 weeks, WT and *Tbr1*^+/−^ mice were euthanised with CO_2_ and transcardially perfused with 20 mL of ice-cold 4% paraformaldehyde (PFA) in 0.1 M phosphate buffer (pH 7.4). The brains were collected and postfixed in 4% PFA for 24 h at 4 °C and then incubated in 30% sucrose in 0.1 M phosphate buffer for 72 h at 4 °C. Coronal brain sections were prepared at 50 µm thickness. Brain sections from 7 animals were used for each group (i.e. WT_30ppm_ vs. *Tbr*^+/−^_30 ppm_ vs. WT_150ppm_ vs. *Tbr*^+/−^_150 ppm_). Brain sections were placed in alcohol/chloroform solution and then rinsed with 70% ethanol, 5 min × 3 times. The sections were then placed in 0.1% Luxol fast blue solution overnight at 56 °C and were destained with 0.05% lithium carbonate solution. Each whole brain section was imaged with a Leica MZ6 stereomicroscope using a Leica Flexacam C1 camera at 1.6X magnification.

### Antibodies and immunohistochemistry

Coronal sections (50 µm) were first permeabilised overnight with 0.25% Triton X-100 in 1 × phosphate-buffered saline (PBST, pH 7.4) at 4 °C. After 3 washes with 1× PBST, non-specific binding was blocked by incubating the sections with 10% normal goat serum (NGS) in 1× PBST for 1 h at RT. The sections were immunolabelled for GluN1 (1:500; AGC-001, Alomone Labs), Shank2 (1:500; 162 204, Synaptic Systems) or Shank3 (1:500; 162 302, Synaptic Systems) and synapsin1/2 (1:500; 106 004, Synaptic Systems) with primary antibody solution prepared in 1× PBST containing 1% NGS for 72 h at 4 °C. The sections were washed in 1× PBST, incubated for 4 h at RT with secondary antibodies (goat anti‐guinea pig IgG‐Alexa Fluor 594, 1:500; A11076, Molecular Probes; goat anti-rabbit IgG-Alexa Fluor 594, 1:500; A11012, Molecular Probes; goat anti‐rabbit IgG‐Alexa Fluor 647, 1:500; Molecular Probes, A21245). No primary antibody controls showed no immunostaining signal for all antibodies used. The sections were further washed in 1× PBST and incubated with Hoechst (Sigma) for 30 min at RT and mounted on microscope slides (Menzel Glaser) in Citifluor mounting medium (Agar Scientific, AF1).

### Confocal imaging and image analysis

GluN1, Shank2 or Shank3 and synapsin1/2 immunostaining in the LA and basal amygdala (BA) was imaged via confocal microscopy (FV1000; Olympus Corporation, Japan) at 63X magnification (UPLSAPO, 1.35 NA) with 3X digital zoom using FluoView 4.0 image acquisition software, yielding images with a size of 512 × 512 pixels (0.138 µm/pixel, both x and y). Images were taken sequentially, and two counts of the line Kalman integration method were applied. For each section, z-stacks were obtained (30–35 images taken at 0.3 µm apart) from a minimum of two regions in the LA and BA per brain slice, and a minimum of three brain slices were imaged per brain. Imaging parameters including laser power, amplifier gain, and offset, were optimised for each antibody and kept consistent for all subsequent imaging to enable direct comparisons between images of different genotype and zinc diet groups. ImageJ software (NIH, USA) was used for puncta-by-puncta and colocalisation analysis. The postsynaptic GluN1, Shank2 or Shank3, and presynaptic synapsin1/2 immunolabelling was corrected for background intensity variation by subtracting a Gaussian (*σ* = 3 pixels) blurred version of the substack. The 3D Objects Counter tool in ImageJ was then utilised to analyse the number and intensity of GluN1, Shank2 or Shank3, and synapsin1/2 puncta and identify their colocalisation in a 3-dimensional space captured by the z-stack. Only GluN1, Shank2 or Shank3, and synapsin1/2 puncta colocalised in each z-plane were counted as colocalised. GluN1, Shank2 or Shank3 puncta that colocalised with synapsin1/2 were defined as synaptic to ensure data are directly relevant to changes occurring at synapses. For each data set, the image analysis criteria including intensity threshold and detection voxel size range were kept consistent for each experimental set, regardless of the genotype and diet manipulation. The intensity and puncta density (number of puncta per 10,000 µm^3^) measurements were compared between different groups by normalising to the average intensity and puncta density values, respectively, calculated from images from the WT 30 ppm dietary zinc group.

### Statistical analysis

All data are presented as mean ± standard error of the mean (SEM). Statistical analyses were conducted using Graphpad Prism 9.0, with a *p* value < 0.05 considered significant. The D'Agostino and Pearson normality test was performed to assess normality and homogeneity of variances in the data. Statistical significance was determined by two-way analysis of variance (ANOVA) with Šídák's *post hoc* test, one-way ANOVA with Tukey’s *post hoc* multiple comparisons, or nonparametric Kruskal–Wallis test, depending on the normality of data distribution and data comparison. Significant results are marked with * = *p* < 0.05, ** = *p* < 0.01, *** = *p* < 0.001, **** = *p* < 0.0001. Details of each statistical test for each data set, experimental 'n' number of neurons, images, and animals are provided in the figure legends.

## Results

To examine the effects of dietary zinc supplementation on ASD-related behaviours and synaptic deficits beyond *Shank3*-ASD mutations, we fed wildtype *Tbr1*^+*/*+^ (WT) and heterozygous mutant *Tbr*^+/−^ mice control (30 ppm) or high (150 ppm) dietary zinc in their normal chow for 6–8 weeks after weaning [45]. This was followed by behavioural testing as well as glutamatergic excitatory synaptic transmission and immunocytochemical analysis in the amygdala at 9–11 weeks of age.

### ***Auditory fear memory deficits in Tbr1***^***+/−***^*** mice are rescued by dietary zinc supplementation***

**Fig. 1:**
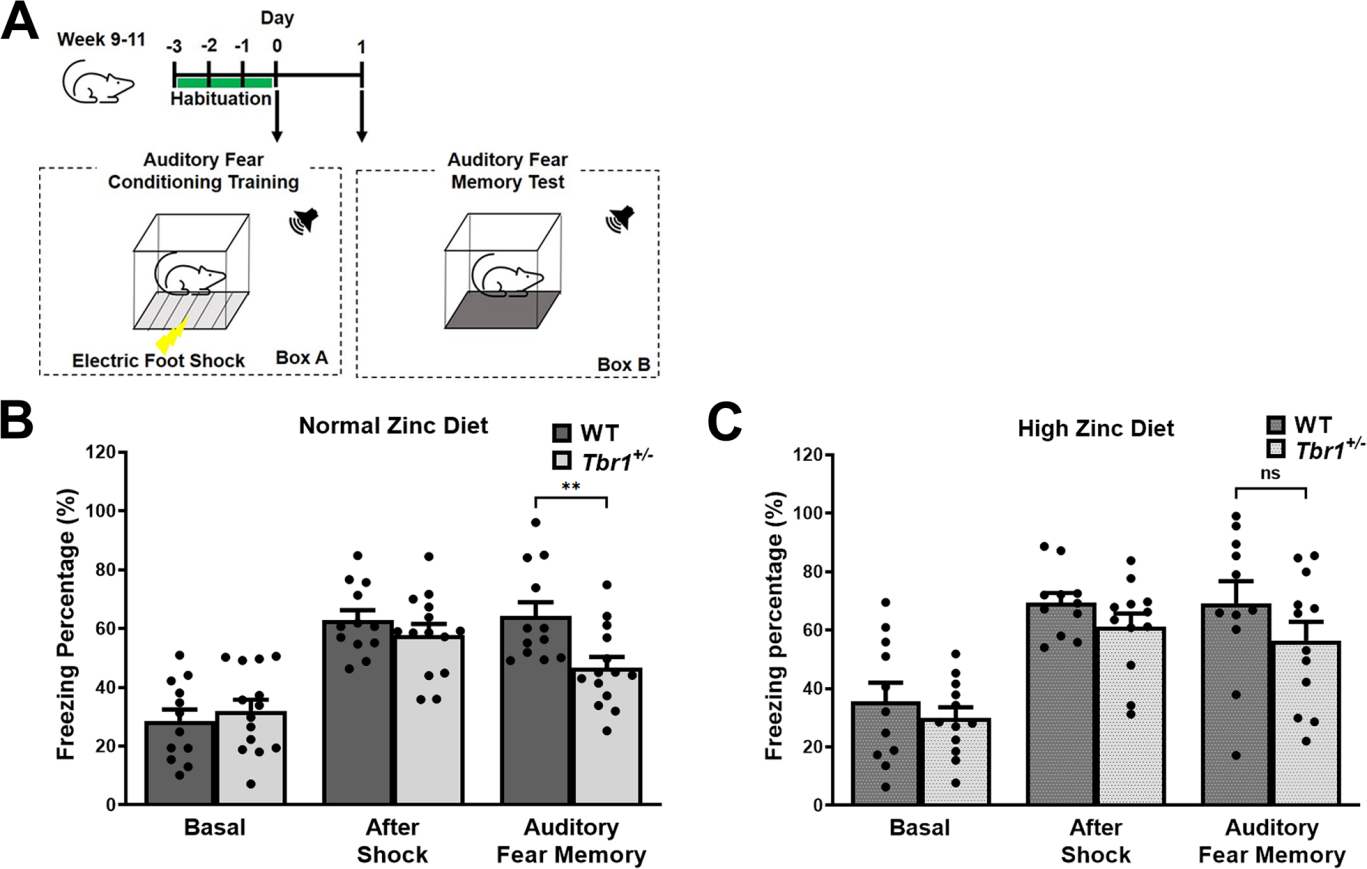
High dietary zinc prevents auditory fear conditioning deficits in the heterozygous *Tbr1*^+/−^ mouse model of autism spectrum disorder (ASD). **A** Schematic of the auditory fear conditioning test. **B** Freezing responses of the wildtype *Tbr1*^+*/*+^ mice (WT) and heterozygous *Tbr1*^+/−^ mice fed with the normal zinc diet (30 parts per million) before electric shock (Basal), immediately after the third electric shock (After Shock), and 1 day after training (Auditory Fear Memory) are shown. *Tbr1*^+/−^ mice (*n* = 14) display impaired auditory fear memory represented by significantly reduced freezing responses compared to WT (*n* = 12). **C** *Tbr1*^+/−^ mice (*n* = 12) fed with high dietary zinc (150 parts per million) no longer display significantly different freezing responses when compared to WT mice (*n* = 11). All data represent mean ± standard error of the mean, analysed using two-way analysis of variance (ANOVA) with Šídák's *post hoc* test and one-way ANOVA with Tukey’s *post hoc* test. ns = not significant, **p < 0.01

Previously it has been shown that one of the profound behavioural deficits observed in mice with haploinsufficiency in *Tbr1* (i.e. *Tbr1*^+/−^) is the impairment in fear conditioning [14]. Therefore, we performed an auditory fear conditioning test to examine the efficacy of our zinc supplementation strategy on this ASD phenotype (Fig. 1A). As expected [14], auditory fear conditioning behaviour was significantly altered in *Tbr1*^+/−^ mice. Specifically, *Tbr1*^+/−^ mice fed the control 30 ppm zinc diet showed a significant deficit in the percentage of time freezing in response to the auditory tone compared with WT mice fed the control 30 ppm diet (WT_30ppm_, 64.27 ± 4.70%; *Tbr*^+/−^_30 ppm_, 46.67 ± 3.64%; WT_30ppm_ vs. *Tbr*^+/−^_30 ppm_, *p* value = 0.0060; Fig. 1B), reflecting a deficit in fear memory. Dietary supplementation with 150 ppm zinc in WT mice did not induce any significant change in fear memory (WT_150ppm_, 69.23 ± 7.48%; Fig. 1C). However, in *Tbr1*^+/−^ mice fed 150 ppm dietary zinc, fear memory was significantly improved, resulting in no significant difference between WT and *Tbr1*^+/−^ mice in fear memory (*Tbr*^+/−^_150 ppm_,56.43 ± 6.44%; WT_150ppm_ vs. *Tbr*^+/−^_150 ppm_, *p* value = 0.2867; Fig. 1C). Increasing dietary zinc can therefore remove the deficit in fear memory in mice expressing the ASD-associated *Tbr1* mutation.

### ***ASD-associated social interaction deficits in Tbr1***^+/−^***mice can be prevented by dietary zinc supplementation***

**Fig. 2:**
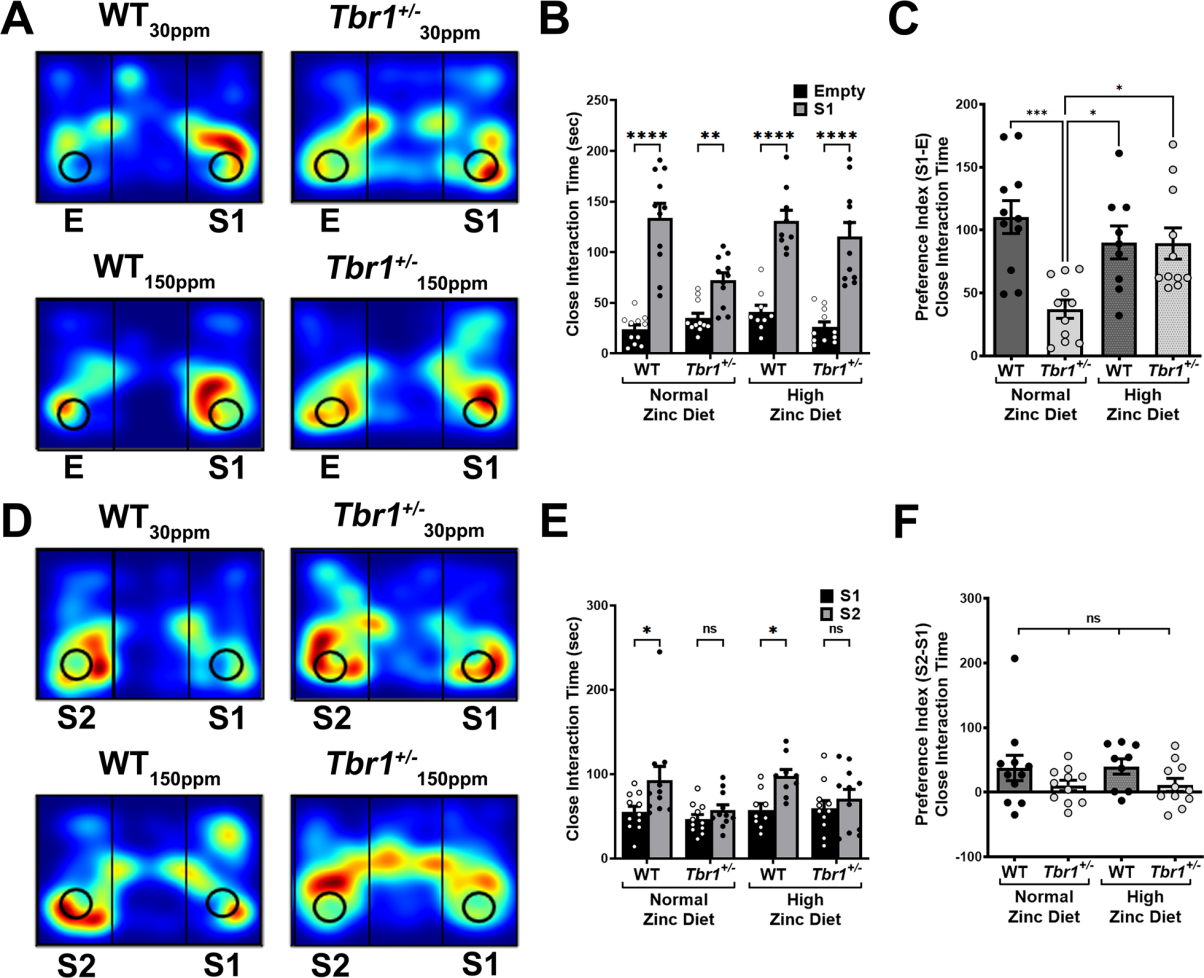
High dietary zinc rescues social interaction deficits in *Tbr1*^+/−^ mice. **A** Heat maps representing example movements of animals during the social interaction test. **B** Both WTmice and heterozygous mutant *Tbr1*^+/−^ mice spend significantly more time closely interacting with stranger 1 mouse (S1) than with the empty cup in the empty chamber (Empty: E), whether fed with the normal zinc diet (30 parts per million) or the high zinc diet. **C** *Tbr1*^+/−^ mice fed the normal zinc diet show a significant deficit in preference index (measured as the difference in time spent between S1 and Empty). The preference index is restored to that of WT mice when the *Tbr1*^+/−^ mice are fed with the high zinc diet. **D** Heat maps representing the example movements of animals during the social novelty test. **E** Dietary zinc supplementation did not rescue social novelty recognition deficit in *Tbr1*^+/−^ mice, measured by the time spent in closely interacting with familiar stranger 1 (S1) mice or novel stranger 2 (S2) mice. **F** The social preference index derived from these results, is not different between WT mice and *Tbr1*^+/−^ mice and this is not changed by dietary zinc supplementation. Each point represents individual mice in each experimental group (Sample size: WT_30ppm_ = 11, *Tbr*^+/−^_30 ppm_ = 11, WT_150ppm_ = 9, *Tbr*^+/−^_150 ppm_ = 11). All data represent mean ± standard error of the mean, analysed using two-way analysis of variance (ANOVA) with Šídák's *post hoc* test and one-way ANOVA with Tukey’s *post hoc* test. ns = not significant, *p < 0.05, **p < 0.01, ***p < 0.005, ****p < 0.001

We also examined the effect of dietary zinc supplementation on ASD-associated social interaction deficits in WT and *Tbr1*^+/−^ mice. We observed no significant phenotypic difference in the social interaction test measured as time spent in close interaction, with both WT and *Tbr1*^+/−^ mice fed the 30 ppm zinc diet both spending significantly more time interacting with the stranger 1 mouse compared to the empty cup in a chamber (WT_30ppm_, empty = 23.82 ± 4.27 s, stranger 1 = 134.09 ± 14.12 s, *p* value  < 0.0001; *Tbr*^+/−^_30 ppm_, empty = 35.00 ± 4.78 s, stranger 1 = 72.09 ± 7.45 s, *p* value  = 0.0091; Fig. 2A and B). Increasing dietary zinc levels to 150 ppm did not alter the social interaction phenotype in either WTor *Tbr1*^+/−^ mice (WT_150ppm_, empty = 40.89 ± 6.65 s, stranger 1 = 131.00 ± 10.45 s, *p* value < 0.0001; *Tbr*^+/−^_150 ppm_, empty = 26.00 ± 5.07 s, stranger 1 = 115.27 ± 14.11 s, *p* value  < 0.0001; Fig. 2A and B). However, *Tbr1*^+/−^ mice fed the control 30 ppm diet did show a deficit in preference index, calculated as the difference in time spent with the stranger mouse and empty cup. Specifically, *Tbr1*^+/−^ mice fed the 30 ppm control zinc diet spent significantly less time with the stranger mouse relative to the exploration time spent closely interacting with the empty cup, compared with WT mice (WT_30ppm_, 110.27 ± 13.03 s; *Tbr*^+/−^_30 ppm_, 37.09 ± 7.28 s; WT_30ppm_ vs. *Tbr*^+/−^_30 ppm_, *p* value  = 0.003; Fig. 2C). Increasing dietary zinc did not alter this time in the WT mice, however high dietary zinc did remove the deficit in *Tbr1*^+/−^ mice such that the preference index was no longer significantly different between WT and *Tbr1*^+/−^ mice (WT_150ppm_, 90.11 ± 13.09 s; *Tbr*^+/−^_150 ppm_, 89.27 ± 12.39 s; WT_150ppm_ vs. *Tbr*^+/−^_150 ppm_, *p* value  > 0.9999; Fig. 2C). Moreover, *Tbr1*^+/−^ mice fed with the 150 ppm zinc diet spent significantly more time with the stranger mouse versus the empty cup than *Tbr1*^+/−^ mice fed with the control 30 ppm zinc diet, demonstrating a zinc diet-induced improvement in preference index (*Tbr*^+/−^_30 ppm_ vs. *Tbr*^+/−^_150 ppm_, *p* value  = 0.0123; Fig. 2C).

Social novelty was then tested in the second phase of the social behaviour test: while the WT mice on the control 30 ppm zinc diet showed a significant difference in interaction time between stranger 1 (i.e. familiar mouse) and stranger 2 (i.e. novel mouse), *Tbr1*^+/−^ mice did not (WT_30ppm_, stranger 1 = 55.09 ± 6.60 s, stranger 2 = 92.64 ± 16.43 s, *p* value  = 0.0254; *Tbr*^+/−^_30 ppm_, stranger 1 = 46.64 ± 5.32 s, stranger 2 = 57.09 ± 6.37 s, *p* value  = 0.8920; Fig. 2D and E). Furthermore, dietary zinc supplementation did not rescue the deficit in time in close interaction with stranger 1 versus stranger 2 mouse in *Tbr1*^+/−^ mice (WT_150ppm_, stranger 1 = 57.56 ± 7.42 s, stranger 2 = 97.33 ± 8.00 s, *p* value  = 0.0345; *Tbr*^+/−^_150 ppm_, stranger 1 = 59.45 ± 9.05 s, stranger 2 = 70.63 ± 11.15 s, *p* value  = 0.8665; Fig. 2D and E). However, the preference index, measured as the difference in time spent with the stranger 1 mouse and stranger 2 mouse, showed no significant difference across the 4 experimental groups (WT_30ppm_, 37.55 ± 19.61 s; *Tbr*^+/−^_30 ppm_, 10.45 ± 8.06 s; WT_150ppm_, 39.78 ± 11.76 s; *Tbr*^+/−^_150 ppm_, 11.18 ± 10.17; Fig. 2F).

### ***Tbr1***^+/−^***mice do not display ASD-related anxiety behaviours nor excessive grooming***

**Fig. 3:**
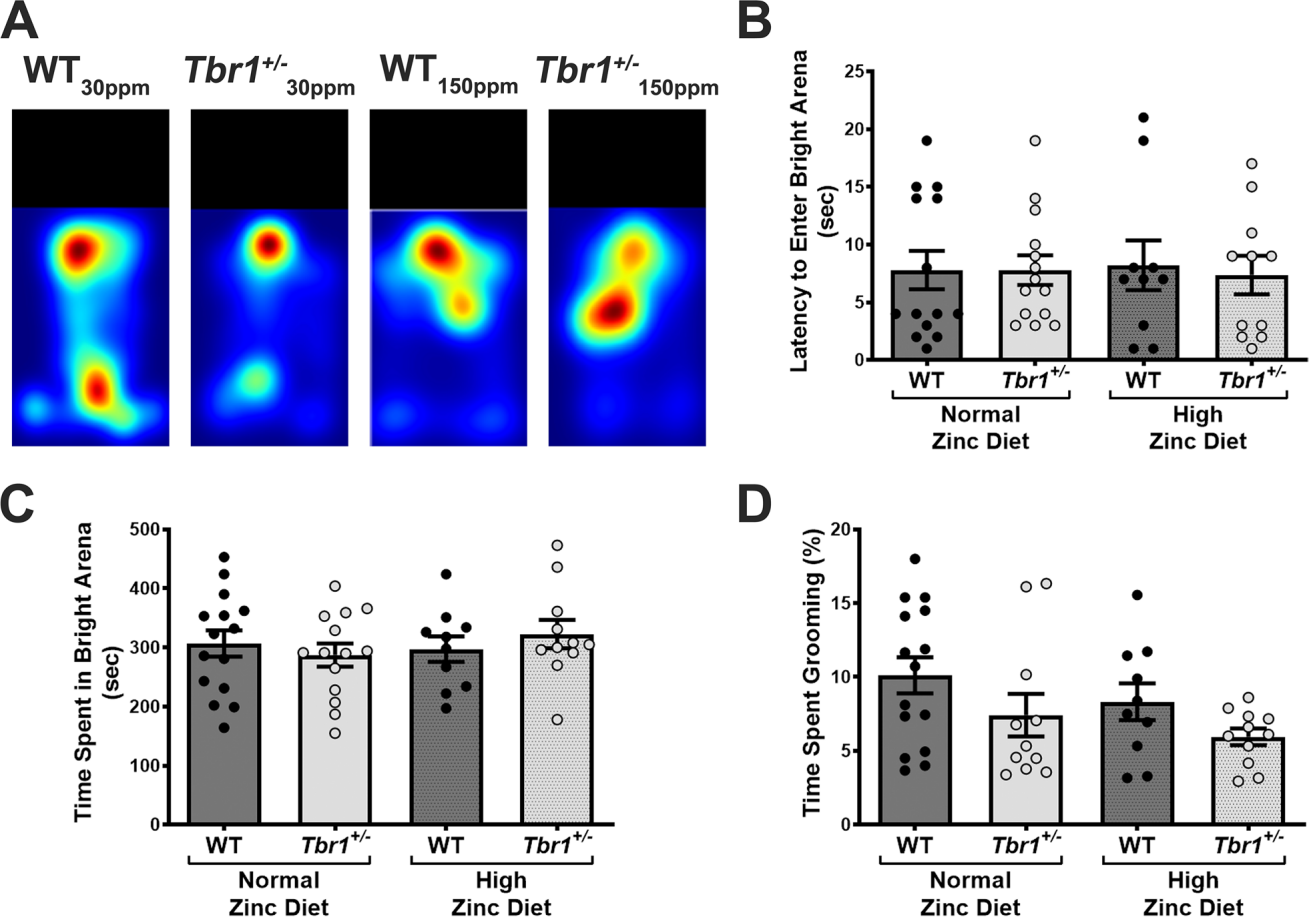
*Tbr1*^+/−^ mice do not display excessive grooming behaviour nor heightened anxious behaviour. **A** Heat maps representing the example movements of animals during the light and dark anxiety test. **B, C** *Tbr1*^+/−^ mice do not show more anxious behaviour than WT mice, indicated by no differences in the latency to enter the bright arena and time spent in the bright arena, and this is not altered by dietary zinc supplementation. (Sample size: WT_30ppm_ = 14, *Tbr*^+/−^_30 ppm_ = 14, WT_150ppm_ = 10, *Tbr*^+/−^_150 ppm_ = 11). **D** *Tbr1*^+/−^ mice do not show excessive grooming behaviour, measured by per cent time spent grooming, and this is not ^+/−^^+/−^ modified by high dietary zinc. (Sample size: WT_30ppm_ = 15, *Tbr*_30 ppm_ = 11, WT_150ppm_ = 10, *Tbr*_150 ppm_ = 11). Each point represents data from individual mice in each experimental group. All data represent mean ± standard error of the mean, analysed using one-way analysis of variance with Tukey’s *post hoc* test

Next, we examined other ASD-associated behaviours such as heightened anxiety traits and excessive grooming in *Tbr1*^+/−^ mice (Fig. 3). The dark–light emergence anxiety test revealed no significant differences between the WT controls and *Tbr1*^+/−^ mice in either the latency to enter the bright arena nor time spent in the bright arena (Latency: WT_30ppm_, 7.79 ± 1.66 s; *Tbr*^+/−^_30 ppm_, 7.79 ± 1.29 s; WT_30ppm_ vs. *Tbr*^+/−^_30 ppm_, *p* value  > 0.9999; Time spent: WT_30ppm_, 306.47 ± 22.28 s; *Tbr*^+/−^_30 ppm_, 287.07 ± 19.49 s; WT_30ppm_ vs. *Tbr*^+/−^_30 ppm_, *p* value  = 0.9078; Fig. 3A–C), and increased dietary zinc did not alter this lack of difference (Latency: WT_150ppm_, 8.20 ± 2.15 s; *Tbr*^+/−^_150 ppm_, 7.36 ± 1.67 s; WT_150ppm_ vs. *Tbr*^+/−^_150 ppm_, *p* value  = 0.9876; Time spent: WT_150ppm_, 297.10 ± 21.65 s; *Tbr*^+/−^_150 ppm_, 322.64 ± 24.02 s; WT_150ppm_ vs. *Tbr*^+/−^_150 ppm_, *p* value  = 0.8760; Fig. 3A–C). Similarly, *Tbr1*^+/−^ mice showed no significant increase in time spent grooming (% time spent grooming: WT_30ppm_, 10.11 ± 1.22%; *Tbr*^+/−^_30 ppm_, 7.41 ± 1.44%; WT_30ppm_ vs. *Tbr*^+/−^_30 ppm_, *p* value  = 0.3558; Fig. 3D), and this was also not significantly altered with the high zinc diet (WT_150ppm_, 8.32 ± 1.25%; *Tbr*^+/−^_150 ppm_, 5.94 ± 0.56%; WT_150ppm_ vs. *Tbr*^+/−^_150 ppm_, *p* value  = 0.5467; Fig. 3D). These results were not surprising as previous work has also described unaltered grooming behaviours and normal anxiety phenotypes measured by open field or elevated plus maze test, respectively, in *Tbr1*^+/−^ mice, when compared to the WT mice [14].

### ***Dysfunctional glutamatergic thalamic-LA synapses in Tbr1***^+/−^***mice are normalised by high dietary zinc***

**Fig. 4:**
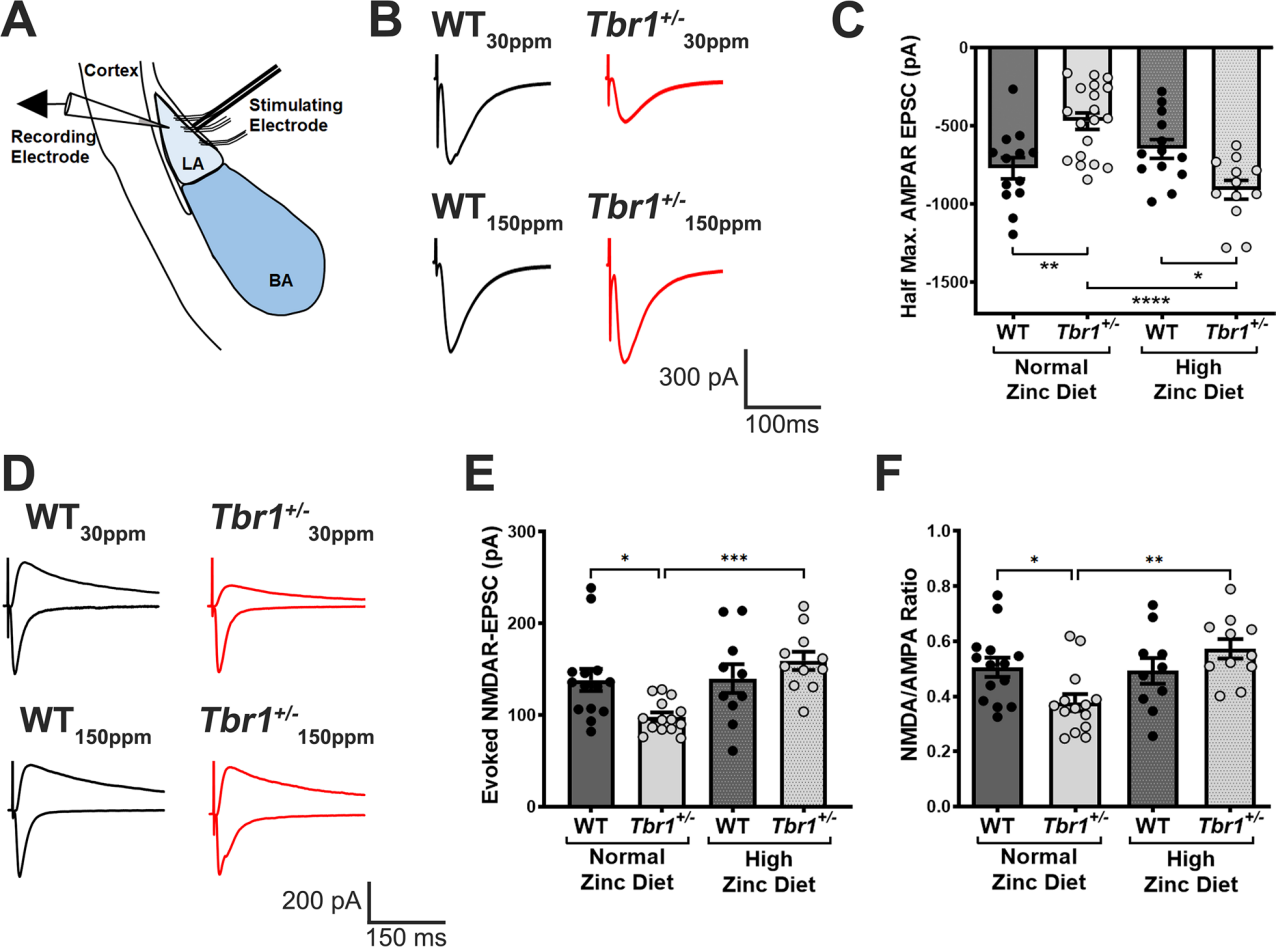
*Tbr1*^+/−^ mice show AMPAR and NMDAR hypofunction at amygdalar thalamic-LA synapses, which are rescued by high dietary zinc. **A** Schematic of electrode placement in the LA for all electrophysiology experiments. LA: lateral amygdala; BA: basal amygdala. **B** Representative traces of half-maximal AMPAR-mediated EPSCs recorded from WT mice and heterozygous mutant *Tbr1*^+/−^ mice fed with normal or high zinc diet. **C** Significantly reduced half-maximal amplitude of AMPAR-mediated EPSCs in *Tbr1*^+/−^ thalamic-LA synapses are restored by high dietary zinc. (Sample size, neurons/animals: WT_30ppm_ = 13/6, *Tbr*^+/−^_30 ppm_ = 19/8, WT_150ppm_ = 13/5, *Tbr*^+/−^_150 ppm_ = 12/6). **D** Representative traces of AMPAR- and NMDAR-mediated EPSCs. **E, F** *Tbr1*^+/−^ mice display significantly impaired NMDAR function, measured by both reduced average amplitudes of evoked NMDAR-mediated EPSCs and reduced NMDAR/AMPAR ratio, which is prevented by dietary zinc supplementation. **E, F** Sample size, neurons/animals: WT_30ppm_ = 14/6, *Tbr*^+/−^_30 ppm_ = 14/6, WT_150ppm_ = 10/5, *Tbr*^+/−^_150 ppm_ = 11/5). Each point represents data from individual neurons recorded in each experimental group. All data represent mean ± standard error of the mean, analysed using one-way analysis of variance with Tukey’s *post hoc* test. ns = not significant, *p < 0.05, **p < 0.01, ***p < 0.005, ****p < 0.001

Auditory inputs from the thalamus and cortex are concentrated in the lateral nuclei of the amygdala (LA) [49], and proper synaptic function onto the pyramidal neurons in the LA is crucial for fear memory [50, 51]. Due to the significant deficit in fear conditioning observed in *Tbr1*^+/−^ mice and its rescue by dietary zinc, we also performed an electrophysiological analysis of excitatory synaptic function in the LA. Specifically, whole-cell patch-clamp recordings of thalamic-LA synapses revealed that excitatory glutamatergic synaptic transmission mediated by AMPARs was significantly decreased in *Tbr1*^+/−^ mice: half-maximal AMPAR EPSC amplitude averaged -771.0 ± 67.94 pA in WT_30ppm_ mice and -469.1 ± 52.92 pA in *Tbr*^+/−^_30 ppm_ mice, with a *p* value of 0.0028 (Fig. 4A–C). Increasing dietary zinc did not significantly alter AMPAR EPSC amplitude in WT mice (WT_150ppm_, -648.0 ± 60.30 pA; WT_30ppm_ vs. WT_150ppm_, *p* value  = 0.5157; Fig. 4A–C). However, dietary zinc supplementation significantly increased half-maximal AMPAR EPSC amplitude in *Tbr1*^+/−^ mice (*Tbr*^+/−^_150 ppm_, -909.2 ± 60.37 pA; *Tbr*^+/−^_30 ppm_ vs. *Tbr*^+/−^_150 ppm_, *p* value  < 0.0001; Fig. 4A–C) so there was no longer a significant difference from WT mice fed with 30 ppm dietary zinc (WT_30ppm_ vs. *Tbr*^+/−^_150 ppm_, *p* value  = 0.4318).

Similarly, we observed that the amplitude of evoked isolated NMDAR-mediated EPSCs, as well as the NMDAR/AMPAR EPSC ratio, were also significantly decreased in heterozygous *Tbr1*^+/−^ mice fed the control zinc diet compared with control *Tbr1*^+*/*+^ mice (NMDAR-EPSC: WT_30ppm_, 138.3 ± 12.11 pA; *Tbr*^+/−^_30 ppm_, 98.35 ± 4.76 pA; WT_30ppm_ vs. *Tbr*^+/−^_30 ppm_, *p* value  = 0.0402; NMDAR/AMPAR ratio: WT_30ppm_, 0.5056 ± 0.0349; Het_30ppm_, 0.3774 ± 0.0308; WT_30ppm_ vs. Het_30ppm_, *p* value  = 0.0498; Fig. 4D–F), revealing a deficit in NMDAR-mediated synaptic transmission in thalamic-LA synapses of *Tbr1*^+/−^ mice [37]. Increasing dietary zinc significantly increased both the amplitude of evoked NMDAR-mediated EPSCs and the NMDAR/AMPAR EPSC ratio in *Tbr1*^+/−^ mice, rescuing the deficit in NMDAR function (NMDAR-EPSC: WT_150ppm_, 139.8 ± 15.70 pA; *Tbr*^+/−^_150 ppm_, 159.3 ± 9.996 pA; *Tbr*^+/−^_30 ppm_ vs. *Tbr*^+/−^_150 ppm_, *p* value  = 0.0003; NMDAR/AMPAR ratio: WT_150ppm_, 0.4922 ± 0.0467; Het_150ppm_, 0.5720 ± 0.0353; Het_30ppm_ vs. Het_150ppm_, *p* value  = 0.0024; Fig. 4D–F). In contrast to the amplitude measurements, no significant differences in the weighted NMDAR decay time were observed between WT and *Tbr1*^+/−^ mice fed with either normal or high dietary zinc (WT_30ppm_, 203.2 ± 31.26 ms; *Tbr*^+/−^_30 ppm_, 162.1 ± 27.97 ms; WT_150ppm_, 247.9 ± 41.53 ms; *Tbr*^+/−^_150 ppm_, 267.9 ± 51.33 ms), suggesting that no change in NMDAR subunit expression occurs in *Tbr1*^+/−^ mice.

### ***High zinc diet supplementation restores presynaptic function at glutamatergic thalamic-LA synapses in Tbr1***^+/−^***mice***

**Fig. 5:**
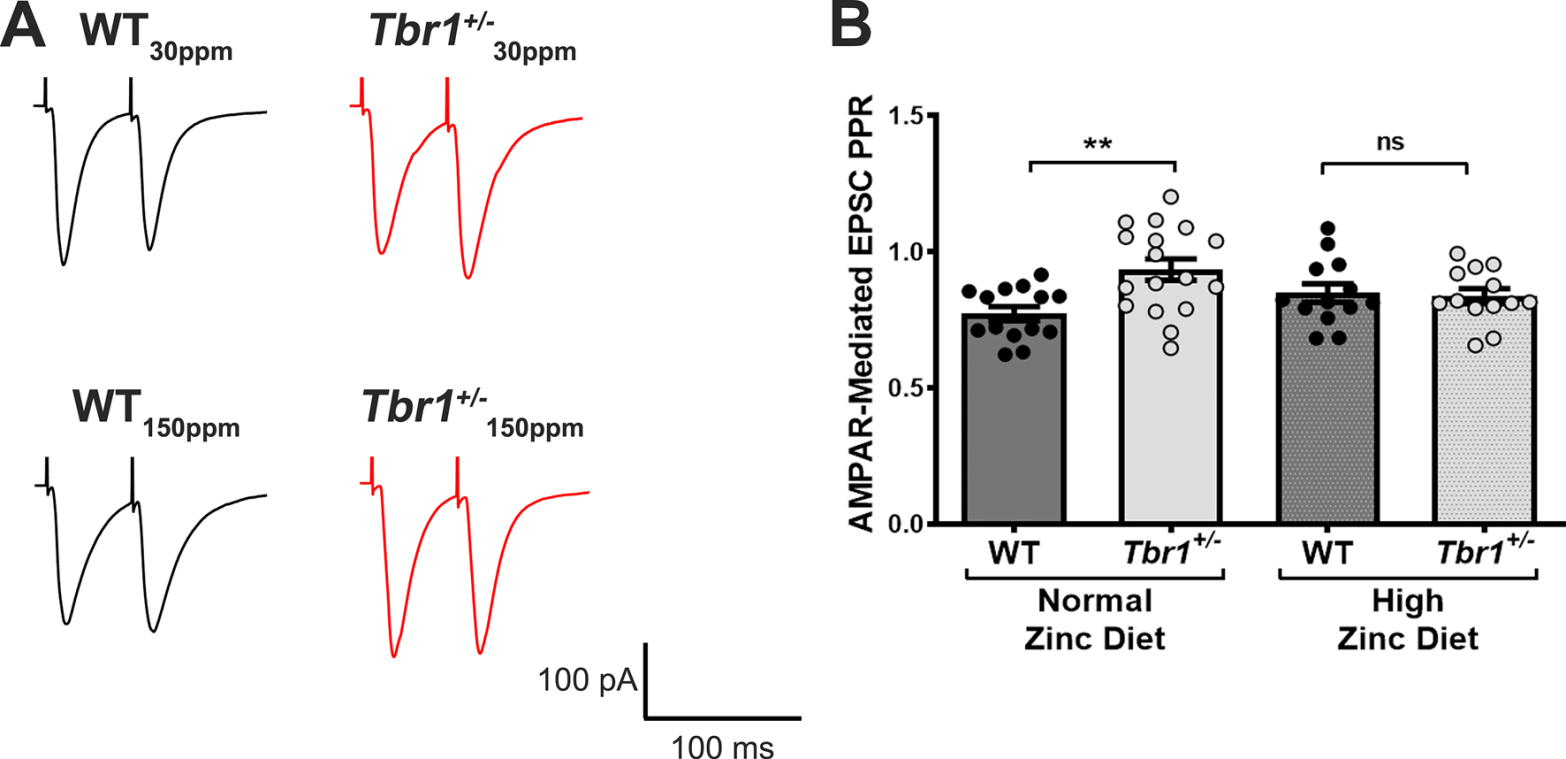
Dietary zinc supplementation normalises the altered paired-pulse ratio (PPR) in *Tbr1*^+/−^ thalamic-LA synapses. **A** Representative traces of AMPAR-mediated EPSC PPR recorded from wildtype *Tbr1*^+*/*+^ mice (WT) and heterozygous mutant *Tbr1*^+/−^ mice fed with normal or high dietary zinc. **B** The altered PPR in *Tbr1*^+/−^ amygdalar thalamic-LA synapses is restored by the high zinc diet. (Sample size, neurons/animals: WT_30ppm_ = 14/6, *Tbr*^+/−^_30 ppm_ = 17/7, WT_150ppm_ = 13/6, *Tbr*^+/−^_150 ppm_ = 13/6). Each point represents individual neurons recorded in each experimental group. All data represent mean ± standard error of the mean, analysed using one-way analysis of variance with Tukey’s *post hoc* test. ns = not significant, **p < 0.01

We also examined AMPAR-mediated paired-pulse ratio (PPR) in principle neurons of the LA to assess presynaptic function in WT and *Tbr1*^+/−^ mice. The ratio of the first to second AMPAR EPSC amplitudes (separated by 50 ms) was measured in all four experimental groups. In the control dietary zinc group, we observed a significant increase in PPR in *Tbr1*^+/−^ mice compared with WT mice (WT_30ppm_, 0.7725 ± 0.0257; *Tbr*^+/−^_30 ppm_, 0.9348 ± 0.0388; WT_30ppm_ vs. *Tbr*^+/−^_30 ppm_, *p* value  = 0.0039; Fig. 5A and B), supporting a lower release probability occurring at thalamic-LA synapses in these ASD mutant mice. Interestingly in mice fed the high zinc diet, there was no significant difference in PPR between *Tbr1*^+/−^ mice compared with WT mice (WT_150ppm_, 0.8486 ± 0.0340; *Tbr*^+/−^_150 ppm_, 0.8370 ± 0.0278; WT_150ppm_ vs. *Tbr*^+/−^_150 ppm_, *p* value  = 0.9953; Fig. 5A and B), showing that increased dietary zinc levels return the PPR to control level, suggesting a return to normal presynaptic function.

### ***High dietary zinc restores synaptic GluN1 and Shank3, but not Shank2, puncta density in the amygdala of Tbr1***^+/−^***mice***

**Fig. 6:**
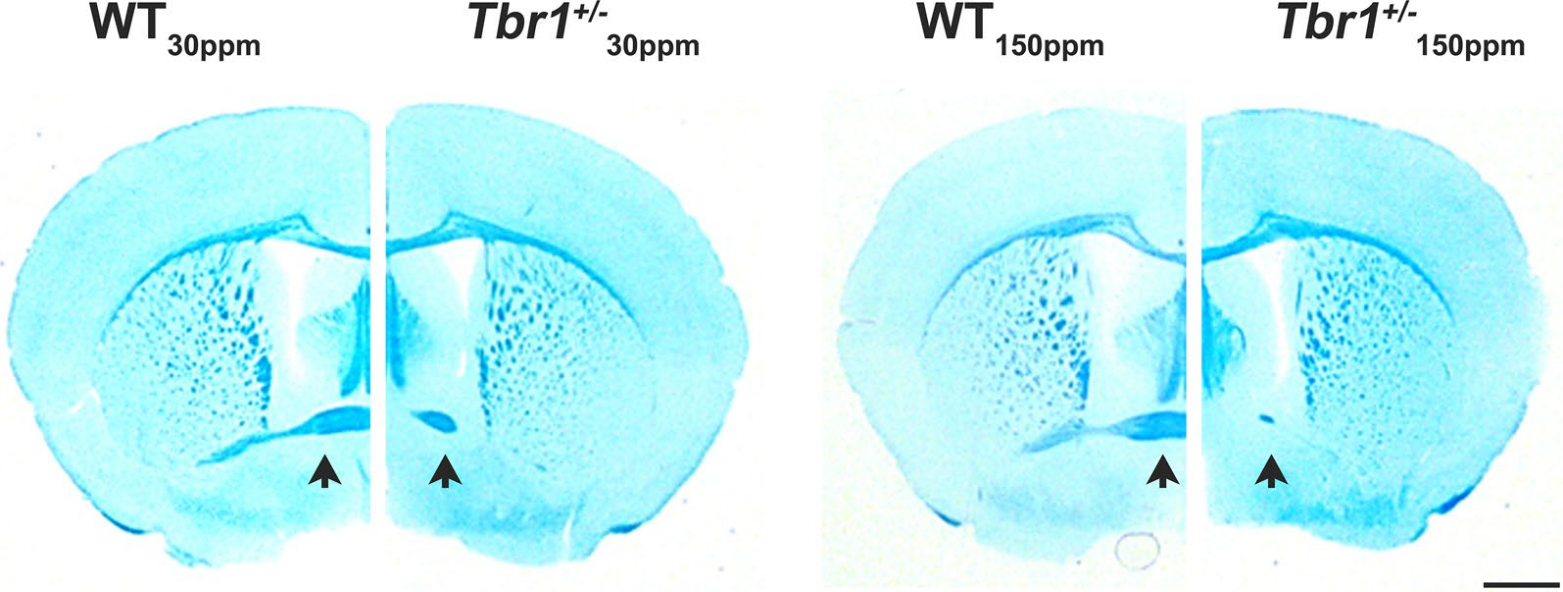
Supplementary dietary zinc does not restore impaired axonal projections in the anterior commissure in *Tbr*^+/−^ mice. Representative images of Luxol fast blue staining performed on brain sections of wildtype *Tbr1*^+*/*+^ mice (WT) and heterozygous mutant *Tbr1*^+/−^ mice fed with normal or high zinc diet (*n* = 7 animals/group). Black arrows indicate inter-amygdalar axonal projections at the anterior commissure. Scale bar = 2.5 mm

**Fig. 7:**
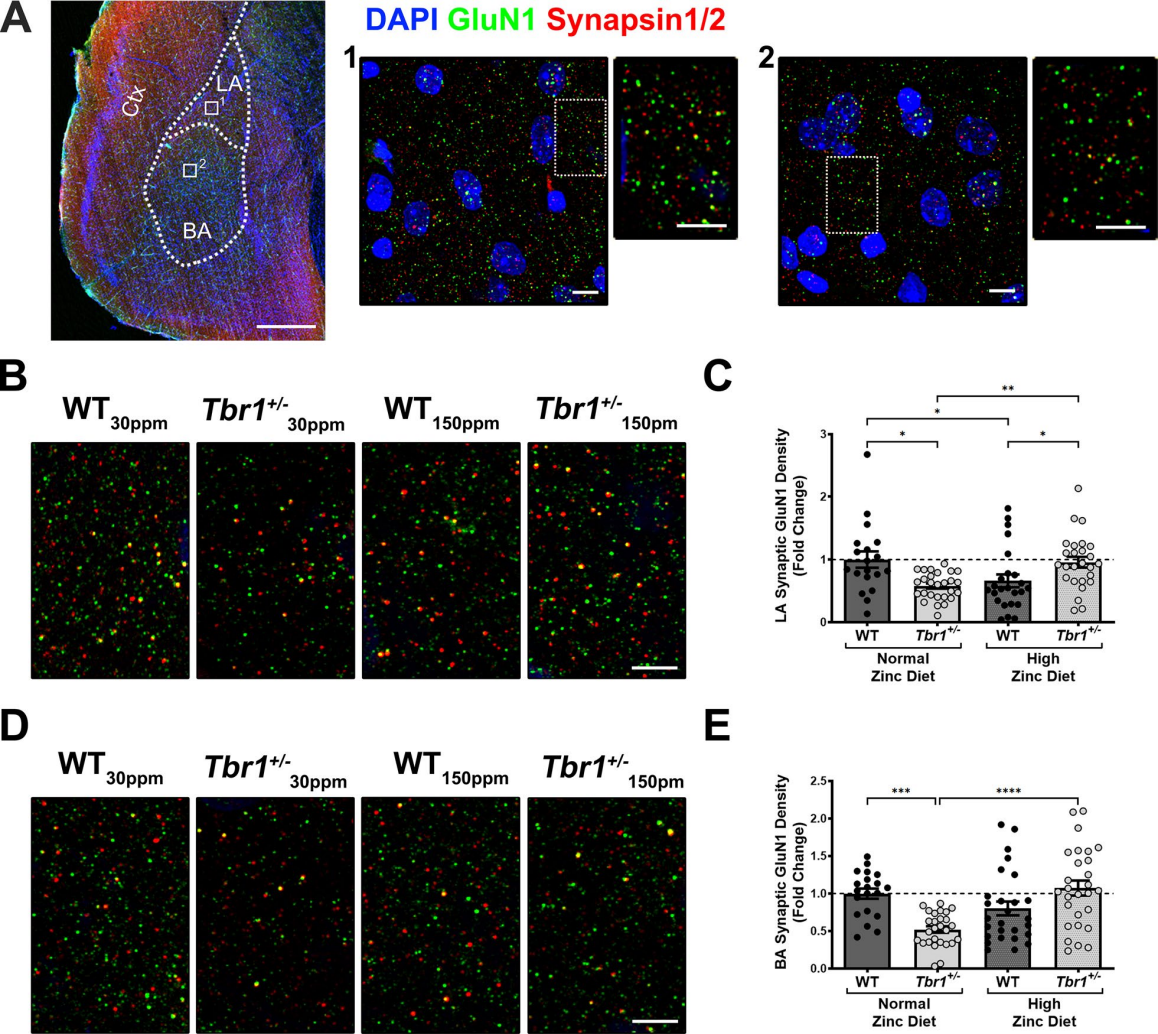
Dietary zinc supplementation restores synaptic GluN1 density in the basal and lateral nuclei of the amygdala (BLA). **A** Representative images displaying amygdala and cortex, labelled with nuclei staining (DAPI: blue), GluN1 (green), and synapsin1/2 (red). Scale bar = 500 µm. Box 1 = example region of interest image taken in the lateral amygdala (LA) and box 2 = example region of interest image taken in the basal amygdala (BA). Scale bar = 10 µm image in the box and 5 µm for zoomed images. **B** Representative images of GluN1 (green) and synapsin1/2 (red) immunolabelling in the LA of WT mice and *Tbr1*^+/−^ mice fed with normal or high zinc diet. **C** Significantly reduced synaptic density of GluN1 in *Tbr1*^+/−^ LA, measured as the density of synapsin1/2-positive GluN1 puncta normalised to the WT_30ppm_ value, is rescued by high dietary zinc. (Sample size, region of interest/animals: WT_30ppm_ = 19/5, *Tbr*^+/−^_30 ppm_ = 27/5, WT_150ppm_ = 24/5, *Tbr*^+/−^_150 ppm_ = 26/5). **D** Representative images of GluN1 (green) and synapsin1/2 (red) immunolabelling in the BA. **E** A reduction in the synaptic density of GluN1 in *Tbr1*^+/−^ BA is prevented by high dietary zinc. (Sample size, region of interest/animals: WT_30ppm_ = 20/5, *Tbr*^+/−^_30 ppm_ = 27/5, WT_150ppm_ = 27/5, *Tbr*^+/−^_150 ppm_ = 28/5). Each point represents an individual region of interest image in each experimental group. Scale bar = 5 µm. All data represent mean ± standard error of the mean, analysed using one-way ANOVA with Tukey’s *post hoc* test. ns = not significant, *p < 0.05, **p < 0.01, ***p < 0.005, ****p < 0.001

To examine whether dietary zinc supplementation normalises thalamic-LA synapse function in *Tbr1*^+/−^ mice via restoration of inter-amygdalar axonal projections, we performed Luxol fast blue staining of brain sections in WT and *Tbr1*^+/−^ mice (*n* = 7/group; example in Fig. 6). Luxol fast blue staining clearly showed the significant reduction in inter-amygdalar axonal projections in *Tbr1*^+/−^ mice, however we observed no improvement in these impaired axonal projections in *Tbr1*^+/−^ mice with the increase in dietary zinc (Fig. 6). Next, we performed immunocytochemical analysis of the GluN1 subunit of the NMDAR in the LA to validate whether changes in the synaptic expression of NMDARs contribute to our behavioural and electrophysiological differences observed in WT and *Tbr1*^+/−^ mice (Fig. 7A–E). Supporting our electrophysiological data (Fig. 4), we observed that synaptic NMDARs, as measured by colocalised GluN1 and synapsin1/2 puncta, were significantly decreased in the LA of *Tbr1*^+/−^ mice compared with WT mice fed with normal 30 ppm zinc diet (WT_30ppm_, 1.0000 ± 0.1304; *Tbr*^+/−^_30 ppm_, 0.5776 ± 0.0413; WT_30ppm_ vs. *Tbr*^+/−^_30 ppm_, *p* value  = 0.0144; Fig. 7B and C). Moreover, high dietary zinc restored the deficits in synaptic GluN1 density in the LA of *Tbr1*^+/−^ mice, such that the synaptic GluN1 densities measured from *Tbr1*^+/−^ mice fed 150 ppm zinc were comparable with WT mice fed with the normal zinc diet (WT_150ppm_, 0.6610 ± 0.1014; *Tbr*^+/−^_150 ppm_, 0.9583 ± 0.0858; *Tbr*^+/−^_30 ppm_ vs. *Tbr*^+/−^_150 ppm_, *p* value  = 0.0041; WT_30ppm_ vs. *Tbr*^+/−^_150 ppm_, *p* value  > 0.9999; Fig. 7B and C). We also conducted immunocytochemical analysis of the GluN1 subunit of the NMDAR in the basal nuclei of the amygdala (BA), the major target for LA pyramidal neurons that are involved in the expression and extinction of conditioned fear memory [52]. As observed in the LA, the synaptic density of GluN1 in mice fed normal dietary zinc levels was significantly reduced in the BA of *Tbr1*^+/−^ mice compared with the WTmice (WT_30ppm_, 1.0000 ± 0.0661; *Tbr*^+/−^_30 ppm_, 0.5206 ± 0.0432; WT_30ppm_ vs. *Tbr*^+/−^_30 ppm_, *p* value  = 0.0015; Fig. 7D and E). In the BA, dietary zinc supplementation also reversed the reduced synaptic GluN1 density in *Tbr1*^+/−^ mice (WT_150ppm_, 0.8009 ± 0.0920; *Tbr*^+/−^_150 ppm_, 1.072 ± 0.0998; *Tbr*^+/−^_30 ppm_ vs. *Tbr*^+/−^_150 ppm_, *p* value  = 0.0015; WT_30ppm_ vs. *Tbr*^+/−^_150 ppm_, *p* value  = 0.8619; Fig. 7D and E).

**Fig. 8:**
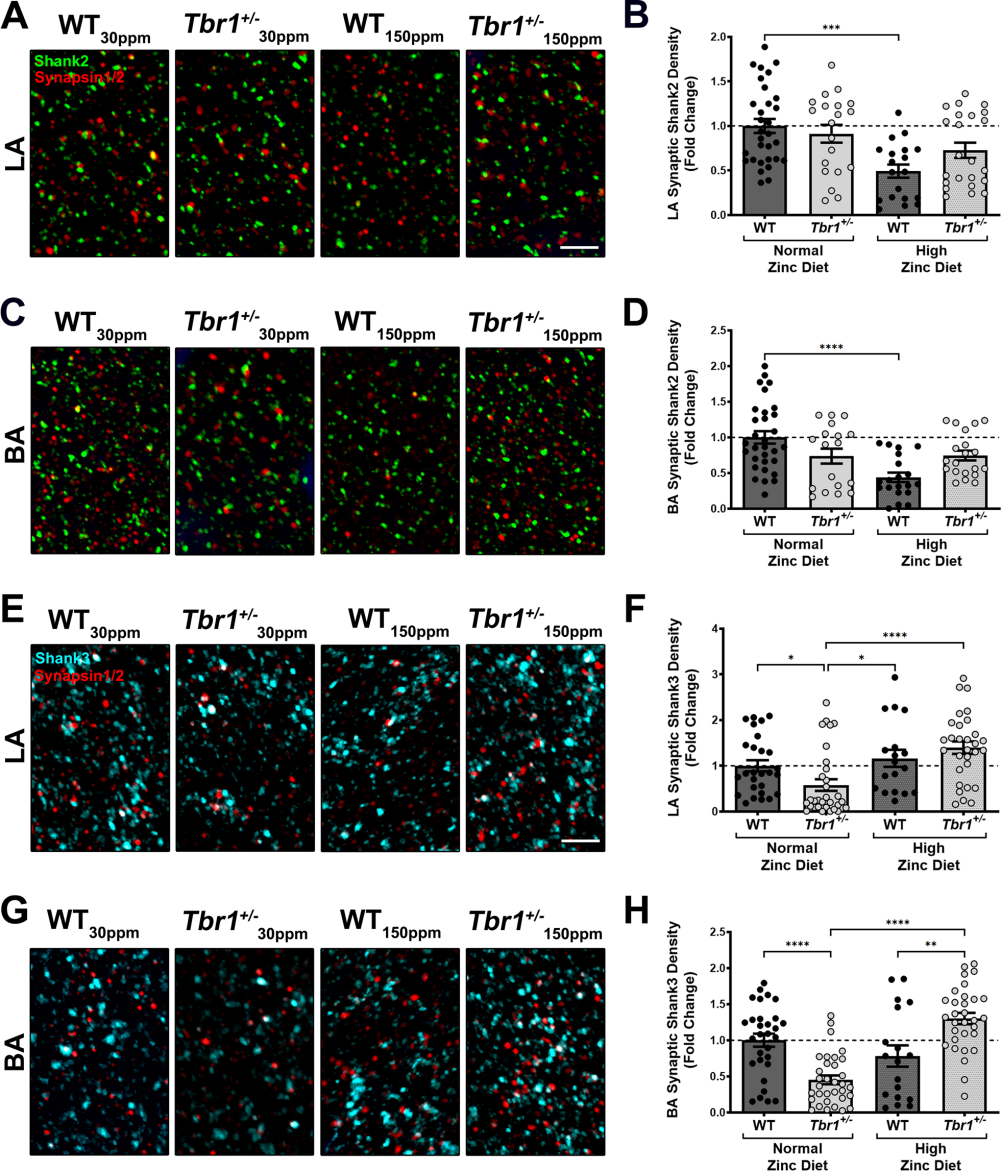
Dietary zinc supplementation restores synaptic Shank3 density, but not synaptic Shank2 density, in the basal and lateral nuclei of the amygdala (BLA). **A** Representative images of Shank2 (green) and synapsin1/2 (red) immunolabelling in the LA of WT mice and *Tbr1*^+/−^ mice fed with normal or high zinc diet. LA: lateral amygdala; BA: basal amygdala. **B** Synaptic density of GluN1 in *Tbr1*^+/−^ LA, measured as the density of synapsin1/2-positive GluN1 puncta normalised to the WT_30ppm_ value, is not significantly different, nor was changed by dietary zinc supplementation (sample size, region of interest/animal number: WT_30ppm_ = 31/5, *Tbr*^+/−^_30 ppm_ = 20/4, WT_150ppm_ = 19/4, *Tbr*^+/−^_150 ppm_ = 23/4). **C** Representative images of Shank2 (green) and synapsin1/2 (red) immunolabelling in the BA. **D** No significant change in the synaptic density of Shank2 is observed in BA of *Tbr1*^+/−^ mice fed with either normal or high zinc diet, when compared to WT_30ppm_ (sample size, region of interest/animal number: WT_30ppm_ = 30/5, *Tbr*^+/−^_30 ppm_ = 18/4, WT_150ppm_ = 20/4, *Tbr*^+/−^_150 ppm_ = 20/4). **E** Representative images of Shank3 (cyan) and synapsin1/2 (red) immunolabelling in the LA. **F** Significantly reduced synaptic density of Shank3 in *Tbr1*^+/−^ LA is rescued by high dietary zinc (sample size, region of interest/animal number: WT_30ppm_ = 27/5, *Tbr*^+/−^_30 ppm_ = 30/5, WT_150ppm_ = 18/4, *Tbr*^+/−^_150 ppm_ = 30/5). **G** Representative images of Shank3 (cyan) and synapsin1/2 (red) immunolabelling in the BA. **H** Reduced synaptic density of GluN1 in *Tbr1*^+/−^ BA is restored by high zinc diet (sample size, region of interest/animal number: WT_30ppm_ = 30/5, *Tbr*^+/−^_30 ppm_ = 33/5, WT_150ppm_ = 18/4, *Tbr*^+/−^_150 ppm_ = 31/5). Each point represents an individual region of interest image in each experimental group. Scale bar = 5 µm. All data represent mean ± standard error of the mean, analysed using nonparametric Kruskal–Wallis test. ns = not significant, *p < 0.05, **p < 0.01, ***p < 0.005, ****p < 0.001

As Shank2 and Shank3 proteins are highly regulated by zinc, and play a critical role in driving postsynaptic glutamate receptor recruitment to the synapse, we also examined the synaptic localisation of Shank2 and Shank3 in WT and *Tbr1*^+/−^ mice fed normal and high dietary zinc. We observed no significant differences in Shank2 expression in either genotype with dietary zinc in either in LA or BA (Shank2 LA: WT_30ppm_, 1.0000 ± 0.0785; *Tbr*^+/−^_30 ppm_, 0.9117 ± 0.1000; WT_150ppm_, 0.4924 ± 0.0744; *Tbr*^+/−^_150 ppm_, 0.7267 ± 0.0851; WT_30ppm_ vs. *Tbr*^+/−^_30 ppm_, *p* value  > 0.9999; *Tbr*^+/−^_30 ppm_ vs. *Tbr*^+/−^_150 ppm_, *p* value  = 0.9509; WT_30ppm_ vs. *Tbr*^+/−^_150 ppm_, *p* value  = 0.1723; Fig. 8A and B. Shank2 BA: WT_30ppm_, 1.0000 ± 0.0867; *Tbr*^+/−^_30 ppm_, 0.7369 ± 0.1042; WT_150ppm_, 0.4391 ± 0.0653; *Tbr*^+/−^_150 ppm_, 0.7453 ± 0.0684; WT_30ppm_ vs. *Tbr*^+/−^_30 ppm_, *p* value  = 0.4383; *Tbr*^+/−^_30 ppm_ vs. *Tbr*^+/−^_150 ppm_, *p* value  > 0.9999; WT_30ppm_ vs. *Tbr*^+/−^_150 ppm_, *p* value  = 0.6654; Fig. 8C and D). However, interestingly we did observe a significant increase in Shank3 synaptic expression in both the LA and BA in *Tbr1*^+/−^ mice fed high dietary zinc, such that Shank3 levels were returned to WT levels (Shank3 LA: WT_30ppm_, 1.0000 ± 0.1209; *Tbr*^+/−^_30 ppm_, 0.5783 ± 0.1266; WT_150ppm_, 1.164 ± 0.1870; *Tbr*^+/−^_150 ppm_, 1.396 ± 0.1357; WT_30ppm_ vs. *Tbr*^+/−^_30 ppm_, *p* value  = 0.0241; *Tbr*^+/−^_30 ppm_ vs. *Tbr*^+/−^_150 ppm_, *p* value  < 0.0001; WT_30ppm_ vs. *Tbr*^+/−^_150 ppm_, *p* value  = 0.6833; Fig. 8E and F; Shank3 BA: WT_30ppm_, 1.0000 ± 0.08972; *Tbr*^+/−^_30 ppm_, 0.4526 ± 0.0610; WT_150ppm_, 0.7823 ± 0.1476; *Tbr*^+/−^_150 ppm_, 1.301 ± 0.0790; WT_30ppm_ vs. *Tbr*^+/−^_30 ppm_, *p* value  < 0.0001; *Tbr*^+/−^_30 ppm_ vs. *Tbr*^+/−^_150 ppm_, *p* value  < 0.0001; WT_30ppm_ vs. *Tbr*^+/−^_150 ppm_, *p* value  = 0.0621; Fig. 8G and H). Together these data show that a deficit in synaptic NMDAR expression occurs in both the basal and lateral nuclei of the amygdala (BLA) in *Tbr1*^+/−^ mice which likely underpins the decrease in NMDAR-mediated currents and the NMDAR/AMPAR ratio, and that this deficit can be rescued by dietary zinc supplementation in *Tbr1*^+/−^ mice, in part, through Shank3-related mechanism.

## Discussion

Here, we show that dietary zinc supplementation prevents impairments in auditory fear memory and social interaction, but not social novelty, in the *Tbr1*^+/−^ mouse model of ASD. At the synaptic level, dietary zinc supplementation also reversed AMPAR and NMDAR hypofunction and normalised presynaptic function at glutamatergic thalamic-LA synapses that are crucial for auditory fear memory [49, 50], and restored synaptic density of the GluN1 subunit essential for functional NMDARs. Together, our data further the understanding of the molecular mechanisms underlying the effect of dietary zinc supplementation and verify the efficacy and breadth of its application as a potential treatment strategy for ASD.

Zinc supplementation is a viable therapeutic approach beyond Shank mutations for ASD phenotypes stemming from the amygdala.

The concept of zinc supplementation as a potential ASD treatment has been based on observations that (1) reduced serum zinc levels have been identified in individuals with ASD [53–57], and (2) prenatal zinc-deficient animals display ASD-like behaviours including increased anxiety, social interaction deficits and altered ultrasonic vocalisation [58, 59]. The efficacy and mechanistic underpinnings of zinc supplementation as a treatment strategy have so far been primarily examined in rodent models carrying ASD-associated *SHANK2* or *SHANK3* mutations, but also in the maternal immune activation model of ASD [37, 41, 44, 45, 59–62]. A focus on *SHANK* models is likely due to (1) the high prevalence of *SHANK2* and *SHANK3* mutations in people affected by ASD [63]; (2) SHANK2 and SHANK3, which are excitatory glutamatergic postsynaptic proteins, contain a SAM domain that directly binds zinc [41, 43, 64]; (3) synaptic localisation and expression of SHANK2 and SHANK3 are highly dependent on zinc [42–44, 64–66]; and (4) zinc deficiency dysregulates the postsynaptic SHANK scaffold, which is thought to contribute to the synaptic mechanisms underlying behavioural deficits in ASD [41, 44, 58]. The data described in the current study is one of the few that have identified zinc supplementation as a viable therapeutic approach beyond ASD-associated *Shank* mutations and provided potential mechanisms underlying its beneficial effect [37, 67], specifically in the amygdala. Together with previous studies [37, 41, 44, 45, 59–62], this shows impairment of zinc sensitive pathways may be a shared biological substrate for ASD behaviours across several ASD models.

The amygdala forms intricate neural circuits with other brain regions such as the prefrontal cortex, thalamus, hypothalamus, hippocampus, and striatum, and plays a principal role in emotional memory and social behaviours [68, 69]. Defective inter- and intra-amygdalar axonal projections and impaired neuronal activation in the amygdalae are major phenotypes observed in *Tbr1*^+*/−*^ mice, and consistent with this, amygdala-dependent behaviours such as social interaction, social novelty recognition, and auditory fear memory are impaired in *Tbr1*^+/−^ mice [14, 37]. In particular, thalamic-LA synapse function is significantly impaired, likely contributing to auditory fear memory deficits observed in *Tbr1*^+/−^ mice. We observed that the amplitudes of both AMPAR- and NMDAR-mediated EPSCs were decreased, and that presynaptic function (as measured by PPR) was altered at thalamic-LA synapses in *Tbr1*^+/−^ mice. Also, the synaptic puncta density of GluN1 and Shank3, was reduced in *Tbr1*^+/−^ amygdalae, indicating that diminished synaptic expression of Shank3 proteins and NMDARs contributes to NMDAR hypofunction at *Tbr1*^+/−^ thalamic-LA synapses. A critical result is that dietary zinc supplementation can restore excitatory glutamatergic pre- and postsynaptic function at *Tbr1*^+/−^ thalamic-LA synapses, as well as the synaptic GluN1 and Shank3 density, comparable to that of WT mice. Together our data suggest that normalisation of thalamic-LA synapse function in *Tbr1*^+/−^ mice is a central mechanism behind the dietary zinc-induced prevention of auditory fear memory impairment.

### Potential zinc-related pathways involved

The majority of zinc ions are extensively bound within proteins as structural or catalytic cofactors in the brain, while a pool of free (or chelatable) zinc is highly localised within synaptic vesicles, accumulated by the vesicular zinc transporter ZnT3, at glutamatergic nerve terminals [40, 70, 71]. In the amygdala, only the excitatory cortical-amygdalar synapses implicated in auditory fear conditioning contain vesicular zinc. In contrast, thalamic projections to the LA involved in auditory fear conditioning lack free zinc, as demonstrated by minimal expression of the *ZnT3* gene [72]. Therefore, the high dietary zinc-induced restoration of glutamatergic thalamic-LA synapse function in the *Tbr1*^+/−^ mice is unlikely to be caused by the enrichment of free zinc, but rather by changes in the availability of protein-bound zinc at these synapses. Further analysis measuring total zinc levels by inductively coupled plasma mass spectrometry, and free or intracellular zinc with zinc indicators (e.g. TFL-Zn or ZnAF-2DA, respectively) [37] would be required in the amygdala to assess this possibility.

At excitatory glutamatergic synapses, bound zinc maintains the organisation of a complex assembly of postsynaptic density proteins where it associates with SAP102, SHANK2, and SHANK3 [43, 64, 73]. The exact role of the zinc-binding motif of SAP102 remains elusive [73], although it may link to its trafficking of AMPARs and NMDARs during synaptogenesis to regulate synaptic organisation and plasticity [74–76]. Conversely, much more is known about zinc’s role in driving synaptic localisation and expression of SHANK2 and SHANK3, and subsequently AMPARs and NMDARs [41, 42, 44, 45, 59, 77, 78]. In *Cttnbp2*^*−/−*^ ASD mice (independent of *Shank* ASD mutations), zinc supplementation induced a significant increase in synaptic expression of SHANK2 and SHANK3, as well as NMDAR (GluN1 and GluN2B) [67]. Our observed zinc-induced rescue of glutamatergic receptor function and GluN1 puncta density in *Tbr1*^+/−^ mice appears to be SHANK3, but not SHANK2, related, suggesting that dietary zinc-induced increases in SHANK3 can drive NMDAR expression at synapses in *Tbr1*^+/−^ mice. Moreover, SHANK3 can regulate glutamatergic presynaptic function via trans-synaptic signalling through the neurexin–neuroligin cell adhesion molecules to recruit presynaptic structural proteins and increase transmitter release, which could further contribute to our observed dietary zinc-related changes in presynaptic and postsynaptic function in *Tbr1*^+/−^ mice [79]. In addition, zinc-binding proteins are located in the presynapse, including Piccolo, Bassoon, Munc13, and RIM, that control the priming, tethering, and exocytosis of synaptic vesicles, and thereby determine presynaptic release probability [80–82]. Dietary zinc supplementation may drive zinc-binding to presynaptic proteins together with zinc-dependent recruitment of SHANK protein complexes pre- and postsynaptically to modify vesicular release probability and prevent abnormal PPR observed in the *Tbr1*^+/−^ mice. Altogether, the supplemented zinc may exert its therapeutic effect through association with zinc-responsive proteins at both pre- and postsynapses that synergistically work together to repair glutamatergic thalamic-LA synapse structure and function.

### Timing and duration are critical in dietary zinc supplementation.

In *Tbr1*^+/−^ mice, dietary zinc supplementation prevents impairments in auditory fear memory and social interaction, but not social novelty recognition impairments. This selective therapeutic effect of dietary zinc supplementation may be attributed to (1) differences in brain circuitry and regions involved in distinct social behaviours [83, 84], (2) discrete zinc responsiveness in different neural pathways [72, 85], and (3) timing of zinc supplementation [86]. The detrimental behavioural phenotypes induced by *Tbr1* haploinsufficiency likely manifest from early stages in brain development [17, 20, 24–26]. Indeed we have recently shown that both social interaction and social novelty deficits are present in *Shank3*^*−/−*^ mice as early as 3 weeks of age, indicating that the neural circuitry responsible for social behaviours is established before weaning [46]. In the current study, dietary zinc supplementation was performed post-weaning and our data suggest this is after the “window” to modulate this circuitry has closed. Indeed, our Luxol fast blue staining supports that some structural deficits, such as in the anterior commissure, are not rescued by dietary zinc supplementation. Similarly adult restoration of *Shank3* expression in 2- to 4.5-month-old *Shank3*^−/−^ mice only partially prevented ASD-associated behavioural phenotypes [87], and clioquinol-induced increases in free zinc in 2- to 4-month-old *Shank2*^*−/−*^ mice recovered social interaction deficits but did not restore defective social novelty recognition behaviour nor normalise heightened anxiety [37]. Together with our data, these studies suggest that specific ASD behaviours are more difficult to alter as development and maturation progresses, and therefore the timing of treatment is an essential factor. The underpinning neural pathways do appear to have a window of modulation during early development however, as supported by the intriguing observation that dietary maternal zinc supplementation during pregnancy and lactation can prevent ASD-associated social behaviour deficits developing in 3-week-old *Shank3*^*−/−*^ offspring, and this persists into adulthood [46]. Timing therefore appears to be critical for zinc-based therapies for ASD-associated mutations in genes such as *Tbr1* and *Shanks* that encode proteins involved in early brain development.

Our dietary zinc treatment strategy, in which animals receive dietary zinc in their food for a minimum of 6 weeks [45, 46], induces not only functional but also structural changes at the synaptic level, and effects on ASD behaviours can be observed 2–3 months after zinc supplementation [46]. In contrast, zinc supplementation for a shorter period through the drinking water for 7 days in *Cttnbp2*-deficient mouse does not restore decreased spine density in the hippocampus, and the initial rescue of social interaction behaviours deteriorated 1 week after discontinuation of zinc [67]. Therefore, not only the timing but also the duration of the zinc treatment could be pivotal in the persistence of synaptic remodelling and subsequent behavioural outcomes. Another notable feature of the dietary zinc supplementation approach in this study is that when used at a physiological level (30 ppm or 150 ppm) [88, 89], the increased zinc levels did not influence behaviours in the WT mice. This is consistent with previous findings that WT mice fed with elevated dietary zinc displayed normal behaviours regardless of the timing, duration, or method of delivery [45, 46, 67], even in the presence of small changes at the molecular or synaptic levels.

### Rationale and translational potential of dietary zinc supplementation.

The supplemented dietary zinc level of 150 ppm was used based on our previous studies examining the effects of supplemented zinc diets to ensure a direct comparison was possible across ASD model genotype effects [45, 46]. We have previously identified the large variance in zinc levels in rodent chow ranging from 25 ppm to 120 ppm [45], resulting in a large “normal” range. How this translates to human dosage is difficult to directly compare. ASD children show lower serum zinc levels, and a recent study showed an increase in cognitive-motor performance following daily zinc supplementation for 12 weeks in ASD children with daily zinc dose to each child equal to their body weight in kg plus 15–20 mg [90]. Nevertheless, a conservative approach is required to discover the effective dosage for ASD treatment. The therapeutic window of zinc concentration is narrow, and zinc can influence the metabolism of other metal ions, such as copper [91–95]. Moreover, zinc does not cross the blood–brain barrier (BBB) freely [61], yet the recent development of nano-particle carriers to aid BBB crossover may prove to be encouraging [96, 97]. Together, dietary zinc supplementation has a great therapeutic potential as an ASD treatment strategy, but additional research is required to bring forward safe and effective clinical use.

### Limitations

The therapeutic effects of zinc supplementation have only been evaluated in the mouse models of ASD [37, 45, 46, 64], including *Tbr1*^+/−^ mice in the current study. Whether zinc-based treatment strategies can induce the same beneficial outcomes in human-derived models and ASD patients, that is reversal of ASD-related behaviours through the restoration of synapse function, requires further examination. An additional challenge will be determining whether ASD diagnosis can precede the therapeutic window in humans. Also, the effects of dietary zinc supplementation on neural circuits other than the thalamic-LA projection pathway such as the olfactory system and anterior commissure-related interhemispheric connectivity that have previously been shown to be dysfunctional in *Tbr1*^+/−^ mice [15, 38, 39], or those that may underlie ASD-associated behaviours observed in *Tbr1*^+/−^ mice (i.e. prefrontal cortex and ventral hippocampus for social memory and behaviours) require examination in future studies. We also recognise the current study focussed on the synaptic axis for the therapeutic role of dietary zinc, and it is possible that given the many roles of zinc, that its therapeutic actions are also indirect, for example via gut-brain signalling.

## Conclusion

In summary, here we demonstrate the effectiveness of dietary zinc supplementation in the reversal of ASD-associated synaptic and behavioural deficits in the *Tbr1*^+/−^ ASD rodent model, highlighting the breadth of this treatment strategy beyond *Shank* ASD mutations. Zinc supplementation can restore impaired glutamatergic synapse function and density, particularly in the thalamic-LA projection pathway, and prevent ASD-associated behaviours in *Tbr1*^+/−^ mice. These data improve our understanding of the mechanisms and the potential of dietary zinc supplementation as a treatment strategy for ASD.

## Data Availability

Not applicable.

## Abbreviations

AMPAR: α-Amino-3-hydroxy-5-methyl-4-isoxazolepropionic acid receptor;
ASD: Autism spectrum disorder
BA: Basal amygdala
BLA: Basolateral amygdala
CaMKII: Calcium/calmodulin-dependent protein kinase II
CASK: Calcium/calmodulin-dependent serine protein kinase
CINAP: CASK interacting nucleosome assembly protein
CNQX: 6-Cyano-7-nitroquinoxaline-2,3-dione
EPSC: Excitatory postsynaptic current
HET: Heterozygous
LA: Lateral amygdala
NMDAR: *N*-Methyl-d-aspartate receptor
PPM: Parts per million
PPR: Paired-pulse ratio
TBR1: T-box brain 1
WT: Wildtype
ZnT3: Zinc transporter 3
